# High Doses of Essential Oil of *Croton Zehntneri* Induces Renal Tubular Damage

**DOI:** 10.3390/plants10071400

**Published:** 2021-07-09

**Authors:** Katarine F. Silva, Diogo B. Peruchetti, Gabriela M. Sirtoli, Christina M. Takiya, Ana Acacia S. Pinheiro, José Henrique Leal-Cardoso, Celso Caruso-Neves

**Affiliations:** 1 Instituto de Biofísica Carlos Chagas Filho, Universidade Federal do Rio de Janeiro, Rio de Janeiro 21.941-902, Brazil; katarine_fs@hotmail.com (K.F.S.); dperuchetti@biof.ufrj.br (D.B.P.); gabrielamodenesi@yahoo.com.br (G.M.S.); cmtakiya@gmail.com (C.M.T.); acacia@biof.ufrj.br (A.A.S.P.); 2 Instituto Superior de Ciências Biomédicas, Universidade Estadual do Ceará, Ceará 60.740-000, Brazil; lealcard@gmail.com; 3 Rio de Janeiro Innovation Network in Nanosystems for Health-NanoSAÚDE/FAPERJ, Rio de Janeiro 21.045-900, Brazil; 4 National Institute of Science and Technology for Regenerative Medicine, Rio de Janeiro 21.941-902, Brazil

**Keywords:** *Croton zehntneri*, essential oil, proximal tubule, tubulointerstitial injury, proteinuria, kidney

## Abstract

The essential oil of *Croton zehntneri* (EOCZ) and its major compounds are known to have several biological activities. However, some evidence shows potential toxic effects of high doses of EOCZ (>300 mg/kg) in amphibian and human kidneys. The aim of the present work was to investigate the effects on renal function of EOCZ at 300 mg/kg/day in healthy Swiss mice and a subclinical acute kidney injury (subAKI) animal model, which presents tubule-interstitial injury (TII). Four experimental groups were generated: (1) CONT group (control); (2) EOCZ, mice treated with EOCZ; (3) subAKI; (4) subAKI+EOCZ, subAKI treated simultaneously with EOCZ. EOCZ treatment induced TII measured by increases in (1) proteinuria; (2) cortical tubule-interstitial space; (3) macrophage infiltration; (4) collagen deposition. A decrease in tubular sodium reabsorption was also observed. These results were similar and nonadditive to those observed in the subAKI group. These data suggest that treatment with EOCZ at higher concentrations induces TII in mice, which could be mediated by protein overload in the proximal tubule.

## 1. Introduction

Kidney disease represents a public health problem and is associated with a high rate of mortality and morbidity [1,2,3]. The central role of tubule-interstitial injury (TII) in the development and progression of kidney disease to end-stage renal disease (ESRD) has been highlighted [4,5]. One of the main causes of the development of TII is albumin overload in the proximal tubule (PT) caused by increased filtration at the glomerular membrane [5,6,7]. This process induces functional changes in PT epithelial cells (PTECs), leading to the development of a pro-inflammatory and pro-fibrotic phenotype [7,8,9,10,11,12,13]. In this context, identification of treatments for TII could represent a forward step to halt the progression of renal disease. Several reports have proposed that essential oils, even those used directly from plant extracts, could be used for the treatment of chronic degenerative diseases such as kidney disease.

*Croton zehntneri* Pax et Hoffm. Vel aff. (Euphorbiaceae) is an aromatic bush native to northeastern Brazil, where it is popularly called “canela de cunhã” [14,15,16]. Its leaves are also used to flavor food, and extracts of its bark and leaves are used by the local people to relieve disturbances of the gastrointestinal tract [14,15,16]. The essential oil of *Croton zehntneri* (EOCZ) and its major compounds are known to have antifungal [17], anti-nociceptive [18], anti-inflammatory [19], gastroprotective [20], and anesthetic properties [21]. EOCZ also enhances wound-healing capacity [22]. Despite these beneficial effects, little attention has been given to the possible effects of EOCZ treatment on the kidneys.

In addition to beneficial effects, there are no reports regarding the adverse effects of EOCZ as a result of its indiscriminate use. However, some reports have shown undesirable side effects of EOCZ constituents when isolated [23,24,25,26,27,28]. In a case reported in 1991, urinary abnormalities, including proteinuria, were observed in a 7-month-old child after accidental oral administration of clove oil related to eugenol [23]. Furthermore, estragole and methyl-eugenol induce renal tubules hyperplasia [24,25]. Some works showed that higher doses of 1,8-cineole (500 mg/kg) treated on Wistar rats promoted renal tubular cell detachment [26,27,28]. These results indicate that EOCZ treatment could become a threat to its users. Therefore, the identification of the effects of EOCZ on the structure and function of the kidneys is an important matter. 

In the present study, we investigated the effect of EOCZ treatment in healthy Swiss mice and in a subclinical acute kidney injury (subAKI) animal model which developed TII induced by PT albumin overload [8,9,10,11,12,13]. Swiss mice were treated with 300 mg/kg/day EOCZ, a dose at the upper range of those used pharmacologically in previous studies [19,20]. Our data showed that oral treatment with EOCZ at 300 mg/kg/day induces tubular dysfunction similar to that observed in the subAKI animal model. These results may be the first evidence of an important side effect of oral administration of high doses of EOCZ.

## 2. Results

### 2.1. EOCZ Treatment Induces Changes in Renal Function Parameters

**Figure 1:**
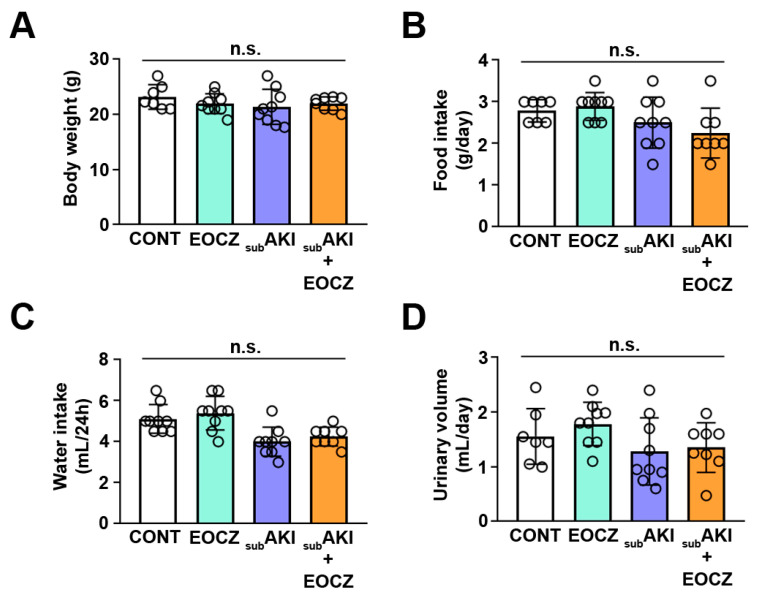
Effect of EOCZ treatment on renal parameters. Mice were randomly divided into four experimental groups as detailed in Section 2: (1) CONT, mice treated with vehicles (white bar, *n* = 7); (2) EOCZ, mice treated with 300 mg/kg/day EOCZ via gavage (green bar, *n* = 9); (3) subAKI, mice treated with 10 g/kg/day BSA via intraperitoneal injection (purple bar, *n* = 9); (4) subAKI and EOCZ, mice treated with BSA and EOCZ (orange bar, *n* = 8). (**A**) Bodyweight; (**B**) food intake, (**C**) water intake, and (**D**) urinary volume. The results are expressed as means ± standard deviation (SD). Dot plots represent the number of individual animals and n.s. represents not significant.

**Figure 2:**
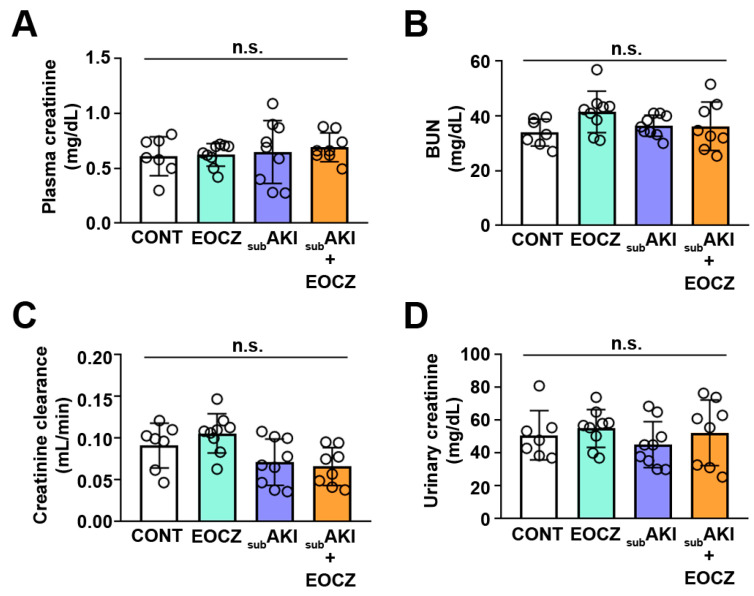
EOCZ treatment did not change glomerular function. Mice were treated as described in Figure 1. The dose of EOCZ used was 300 mg/kg/day, as described in the Material and Methods section. (**A**) Plasma creatinine concentration, (**B**) BUN levels, (**C**) urinary creatinine concentration, and (**D**) creatinine clearance (CCr). Dot plots represent the number of individual animals. Results are expressed as means ± SD and n.s. represents not significant.

To determine if the treatment with EOCZ was beneficial or promoted side effects on renal function, we treated healthy or tubular injured Swiss mice. Four groups were generated as described in the Materials and Methods section (Section 4.3). Bodyweight, food intake, water intake, and urinary output were not changed in all groups studied (Figure 1A–D). Glomerular function assessed by plasma creatine, BUN (blood urea nitrogen), and creatinine clearance, a marker of the glomerular flow rate (GFR), as well as urinary creatinine, were also not changed (Figure 2A–D).

**Figure 3:**
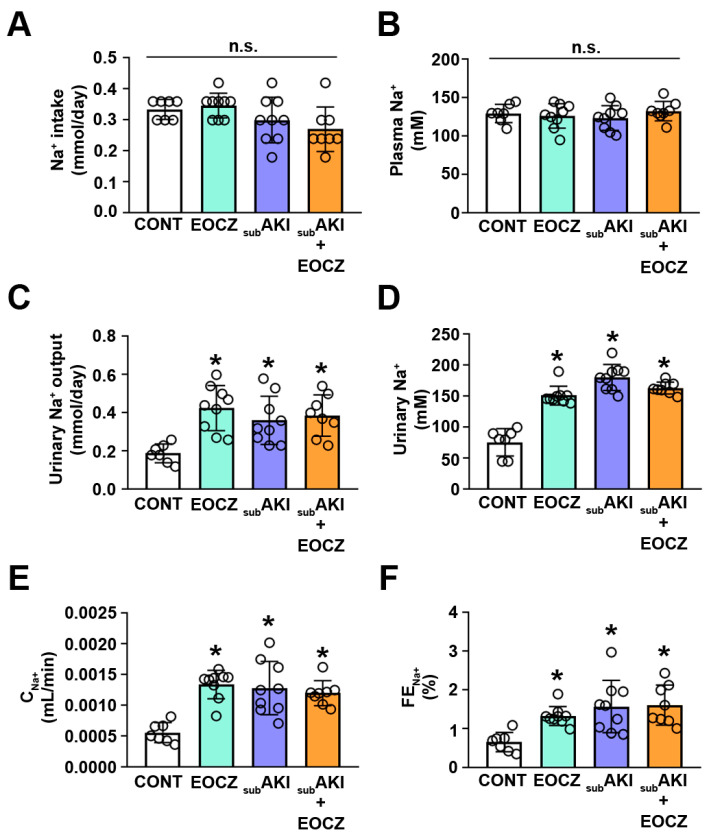
EOCZ treatment alters tubular Na^+^ handling. Mice were treated as described in Figure 1. The dose of EOCZ used was 300 mg/kg/day, as described in the Material and Methods section. (**A**) Na^+^ intake, (**B**) plasma Na^+^ concentration, (**C**) urinary Na^+^ output, (**D**) urinary Na^+^ concentration, (**E**) renal Na^+^ clearance (C_Na+_), and (**F**) fractional excretion of Na^+^ (FE_Na+_). Dot plots represent the number of individual animals. The results are expressed as means ± SD and n.s. represents not significant. * *P* < 0.05 versus CONT group.

In addition, Na^+^ intake and plasma Na^+^ concentration did not change in the EOCZ, subAKI, and subAKI+EOCZ groups (Figure 3A,B). However, urinary Na^+^ output, urinary Na^+^ concentration, and Na^+^ clearance increased in these groups compared with the CONT group, demonstrating possible changes in renal Na^+^ handling (Figure 3C–E). This idea was confirmed by the observation that fractional excretion of Na^+^, a marker of tubular Na^+^ handling, was also increased in the EOCZ, subAKI, and subAKI+EOCZ groups (Figure 3F).

### 2.2. Urinary Protein Excretion Is Modulated by EOCZ Treatment

**Figure 4:**
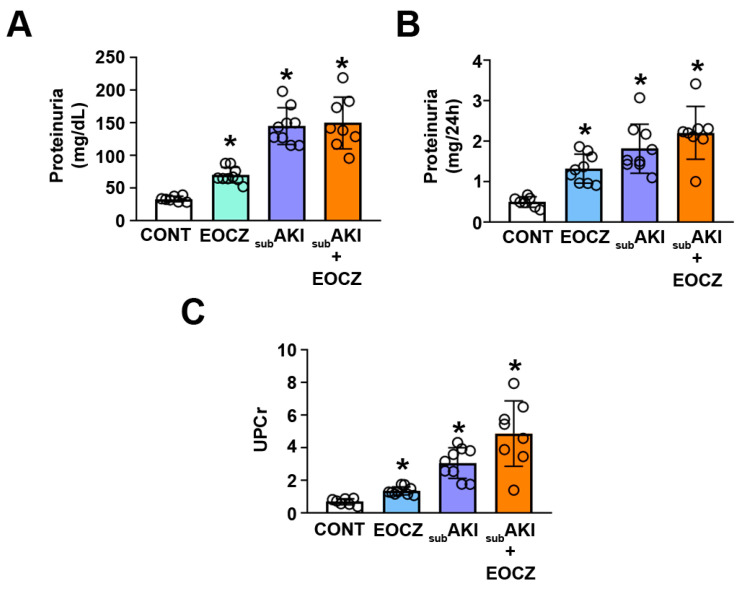
EOCZ treatment induces proteinuria. The dose of EOCZ used was 300mg/kg/day, as described in the Material and Methods section. (**A**) Proteinuria (mg/dL), (**B**) urinary proteins (mg/24 h), and (**C**) ratio of urinary proteins to creatinine (UPCr). Dot plots represent the number of individual animals. The results are expressed as means ± SD. * *P* < 0.05 versus CONT group.

Under physiologic conditions, plasma proteins are filtered in the glomerulus and totally reabsorbed by PTECs [7,29]. On the other hand, renal injuries at glomerular or tubular levels lead to an increase in urinary protein excretion [5,7,11,12,29]. We observed that proteinuria (Figure 4A,B) and the urinary protein/creatinine ratio (Figure 4C) were significantly increased in the EOCZ and subAKI groups. No further increase in these parameters was observed in the subAKI+EOCZ group compared with the subAKI or EOCZ groups individually, but they were increased when compared with the CONT group. 

Since changes were not observed in glomerular function markers (Figure 2) but an increase in FE_Na+_ and proteinuria, we postulated that EOCZ treatment could lead to tubular damage without change in glomerular function. The possible TII induced by EOCZ treatment will be assessed in the next section.

### 2.3. EOCZ Treatment Promotes Tubulointerstitial Injury

**Figure 5:**
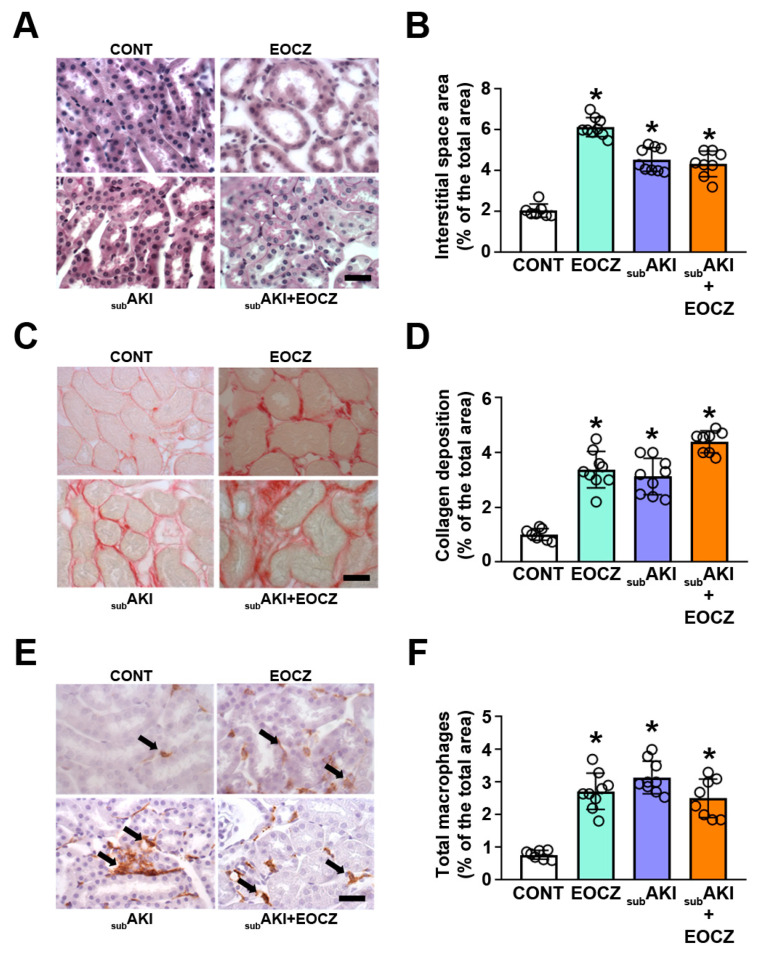
EOCZ treatment induces tubule-interstitial injury. The dose of EOCZ used was 300 mg/kg/day, as described in the Material and Methods section. Representative images are shown in (**A**,**C**,**E**). Scale bar, 40 µm. (**B**,**D**,**F**) The quantification analyses. (**A**,**B**) Area of interstitial space, (**C**,**D**) total collagen deposition, and (**E**,**F**) macrophage infiltration. Black arrows in (**C**) show F4/80-positive cells (murine macrophage marker). Dot plots represent the number of individual animals. The results are expressed as means ± SD. * *P* < 0.05 versus CONT group.

TII involves changes in a tubular structure, immune cell infiltration, and collagen deposition [5,8,9,10,11,12,13,30]. The EOCZ and subAKI+EOCZ groups showed an increase in (1) the area of tubulointerstitial space analyzed by periodic acid-Schiff staining (Figure 5A,B); (2) collagen deposition, analyzed by picrosirius red (Figure 5C,D); (3) macrophage infiltration, measured by F4/80-positive cells (Figure 5E,F). The increase in these parameters was of the same magnitude and nonadditive to those observed in the subAKI group. These data suggest that EOCZ treatment per se induces TII in a similar way to that observed in the subAKI animal model.

## 3. Discussion

Despite the widespread use of EOCZ for the treatment of several diseases [16,19,20,21,31,32], little is known about the effect of EOCZ on renal function. Our findings provide strong evidence that oral treatment with a high dose of EOCZ for seven consecutive days results in undesirable tubular dysfunction, affecting renal sodium and protein handling, which could be associated with the development of cortical TII. These results suggest that indiscriminate use of EOCZ for the treatment of different illnesses is ill-advised without health professional supervision.

The results presented here suggest an upper bound for the dose of EOCZ that should be used in treatments via systemic administration. This is relevant because the pharmacologic studies available in the literature report a large range of doses for the pharmacologic efficacy of EOCZ; for example, 3–30 mg/kg for an anti-inflammatory effect [19] and 3–300 mg/kg for gastroprotection [20]. Thus, for pharmacologic treatment with systemic administration of EOCZ, including for gastric ulcer, the present study suggests that it is advisable to use doses less than 300 mg/kg. In addition, the people in northeast Brazil prepare alcoholic beverages with *C. zehntneri* leaves and small branches. Extraction of EOCZ in the presence of alcohol may result in a high concentration of EOCZ and lead to ingestion of high doses of EOCZ when the beverage is consumed.

Side effects of herbal compounds have been associated with the generation of reactive intermediates by bioactivation processes [24,25]. It has been reported that aristolochic acids, which are present in extensively used herbal medicines, induced tubular atrophy, tubulointerstitial fibrosis, and low-molecular-weight proteinuria as well as urothelial cancer in healthy humans [33,34,35,36]. In addition, chemical analysis of EOCZ revealed that its major compounds, anethole, estragole, eugenol and 1,8-cineole, are metabolized, leading to the production of toxic metabolites that promote tubular damage [20,21,22,26,27,28,31,37,38,39]. In agreement with these observations, we showed that treatment with EOCZ induced macrophage infiltration associated with TII. Thus, it is plausible to imagine that the deleterious effects of oral treatment of EOCZ on the kidneys observed in the present study could be correlated to potentially toxic metabolites. Further experiments are necessary to investigate this issue.

In the present work, we used CCr, plasma creatinine, and BUN as markers of GFR. These parameters have been used in clinical practice as well as in animal models of renal diseases [9,10,11,40]. Urinary flow is another important parameter since it has been reduced in acute kidney injury (AKI) due to an abrupt decrease in glomerular function [41]. In the present work, it was observed that all these parameters, including urinary flow, were not modified by EOCZ treatment. In agreement, similar results have been obtained in the subAKI animal model by different groups [10,11,12,13]. 

On the other hand, it has been shown, in chronic kidney disease (CKD) at end-stage, that CCr is not a good marker of GFR. At this condition, the loss of tubular function leads to the reduction of PT creatinine secretion as well as in creatinine urinary excretion misleading the evaluation of glomerular function [4,42]. Herein, it was observed that urinary creatinine was not changed by EOCZ treatment showing that PT creatinine handling is not modified. Additionally, we observed that plasma BUN was not altered in EOCZ treated animals. Together these results indicate that EOCZ treatment did not alter glomerular function. 

Renal tubular damage could be associated with different causes [4,5]. In the subAKI animal model, it has been proposed that there is hyperfiltration of albumin even though the total GFR does not change [8,9,10,11,12,13]. In these conditions, there is an overload of albumin at the PT, which leads to changes in the albumin endocytic machinery and a decrease in albumin reabsorption, which promotes a further increase in proteinuria [4,5,11,12]. All these processes are associated with pro-inflammatory and pro-fibrotic phenotypes and, consequently, with the development of TII [4,5,11,12]. We observed that treatment with EOCZ induced tubular damage similar to that observed in the subAKI animal model. Particularly, proteinuria, collagen deposition, and macrophage infiltration were observed. The observation that the simultaneous treatment of subAKI mice with EOCZ did not ameliorate the tubular damage and did not promote addictive effects indicates that they share a similar mechanism of development. In agreement, the proteinuria level was of similar magnitude in the EOCZ and subAKI groups.

The kidneys control sodium homeostasis by modulating tubular handling of sodium [43]. In many renal diseases, especially of tubular origin, alterations in tubular reabsorption of sodium are observed with no changes in GFR [30,44,45,46]. Previously, our group showed that higher urinary Na^+^ excretion was associated with a reduction in tubular sodium transporter activity in a subAKI animal model [47]. Here, we observed that treatment with EOCZ similarly increased the fractional excretion of sodium, a marker of tubular sodium reabsorption, without changes in glomerular function. The observation that glomerular function is not altered by EOCZ treatment makes it more difficult to identify. In addition, this process could promote renal scars, which could lead to more severe injuries after a second renal insult. In agreement with this view, it was observed previously that subAKI animals have more severe renal damage when subjected to sepsis induced by cecal ligation and puncture than control animals [8].

In conclusion, treatment with high doses of EOCZ has toxic effects on renal function, leading to TII and proteinuria. Indiscriminate use of essential oils without medical supervision is therefore not advised.

## 4. Materials and Methods

### 4.1. Plant Material

Leaves of *Croton zehntneri* were collected in September 1998 near the city of Viçosa, CE, Brazil. The identification of the plants was confirmed by Dr. F.J. Abreu Matos (Laboratory of Natural Products, Universidade Federal do Ceará). A voucher specimen (no. 277477) was deposited in the herbarium of Prisco Viana, Universidade Federal do Ceará.

### 4.2. Extraction and Chromatographic Analysis of EOCZ

EOCZ was isolated from freshly chopped leaves by steam distillation and chemically analyzed as described previously [16,20,21,22]. Chemical characterization of EOCZ was performed by gas chromatography/mass spectrometry. The chromatographic analysis was carried out on a Hewlett-Packard 6971 chromatograph using the following analytical conditions: a dimethylpolysiloxane DB-1 fused silica capillary column (30 m × 0.25 mm; 0.1 mm); helium (1 mL/min) as carrier gas; 250 °C injector temperature; 200 °C detector temperature; a column temperature of 35–180 °C at 4°C/min and then 180–250 °C at 10 °C/min; and mass spectra with the electronic impact of 70 eV. The compounds were identified using a mass spectral library search and ^13^C-nuclear magnetic resonance spectroscopy. The composition of the essential oil from the leaves of *Croton zehntneri* used in this study has been described previously [20,21,22,31]. Briefly, the composition of EOCZ was 85.7% anethole, 4.8% estragole, 2.95% 1,8-cineole, 2.2% *trans*-caryophyllene, and 2.23% unidentified compounds.

### 4.3. Animals and Experimental Protocol

Male Swiss mice (8 weeks old) were kept at a constant temperature (22 ± 2 °C) in a 12 h/12 h light/dark cycle with free access to standard chow and water. All animal procedures were conducted in accordance with the National Institutes of Health Guide for the Care and Use of Laboratory Animals. All experimental protocols were reviewed and approved by the Institutional Ethics Committee of Universidade Federal do Rio de Janeiro (protocol number 043/18).

The development of the subAKI animal model and EOCZ treatment was performed as previously published [8,9,10,11,12,13,16]. Briefly, mice were divided randomly into 4 different groups: (1) CONT group, mice treated with saline (used as a vehicle for bovine serum albumin (BSA)) via intraperitoneal injection, and water (used as a vehicle for EOCZ) via gavage; (2) EOCZ group, mice treated with EOCZ 300 mg/kg/day via gavage, and saline via intraperitoneal injection; (3) subAKI group, mice treated with BSA 10 g/kg/day via intraperitoneal injection and water via gavage; and (4) subAKI+EOCZ group, mice treated with both BSA and EOCZ. All groups were treated daily for 7 consecutive days.

On day 5 of treatment, the mice were housed in metabolic cages for 48 h. Water, food, and Na^+^ intake were measured, and urine output in the last 24 h was collected. The mice were then euthanized with a mixture of ketamine (240 mg/kg) and xylazine (15 mg/kg). Blood samples were obtained by cardiac puncture. Kidneys were perfused with heparinized saline, removed, and prepared for histologic and immunohistochemical analyses.

### 4.4. Renal Function Analysis

Renal function was analyzed as described previously [8,9,10,11,12,13]. The measurements described below, in plasma and 24 h urine, were performed in our laboratory using commercial kits available. Plasma and urinary Na^+^ levels were determined by the photometric and colorimetric test (Sodium Rapid kit, no. 573351; Human Diagnostics, Wiesbaden, Germany). The urinary protein concentration was determined by the Pyrogallol Red method (Gold Analisa kit no. 498M; Belo Horizonte, MG, Brazil). The plasma and urinary creatinine concentrations were measured using the alkaline picrate method (Gold Analisa kit no. 335). Blood urea nitrogen (BUN) was determined by the urease method (Ureia CE, Labtest kit no. 27-500; Lagoa Santa, MG, Brazil). The following parameters were calculated as described previously by our group [47]: (1) creatinine clearance (CCr); 2) Na^+^ clearance (C_Na+_); (3) fractional excretion of Na^+^ (FE_Na+_); (4) mass of urinary proteins in 24 h (mg/24 h); and (5) ratio between urinary proteins and creatinine (UPCr).

### 4.5. Histologic and Immunohistochemical Analyses

Histologic and immunohistochemical analyses were performed as previously published [8,9,10,11,12,13]. Briefly, the kidneys were fixed in a 4% buffered formalin solution and embedded in paraffin, and histologic sections (3-μm- or 7-µm-thick) were obtained. The 3-μm-thick kidney sections were stained with periodic acid-Schiff reagent (Sigma-Aldrich, St Louis, MA) for assessment of the area of interstitial space. A specific antibody (dilution 1:50, cat MCA497; AbD Serotec, Raleigh, NC) was used to determine F4/80-positive cells (macrophages) in the renal cortex [8,9,10,11,12,13]. Sections (7-µm-thick) were stained with picrosirius red to determine the deposition of total collagen in the renal cortex. All images were acquired using a Nikon 80i Eclipse microscope (Nikon, Japan), and 15 randomly selected fields from each animal were analyzed with Image-Pro Plus software version 7.0.1.658 (Media Cybernetics, Rockville, MD, USA). The area of interstitial space was calculated as a percentage of the total tissue area. Macrophage infiltration was calculated as the percentage of positive staining in relation to the total tissue area. Cortical collagen deposition was quantified as a percentage of positive red staining fibers in relation to the total tissue area. All analyses were conducted in a blinded manner.

### 4.6. Statistical Analysis

All data are expressed as means ± standard deviation (SD). Graphics and statistical analyses were performed using GraphPad Prism 8 (version 8; GraphPad Software, San Diego, CA; www.graphpad.com). Statistical differences among the experimental groups were determined by a one-way analysis of variance test followed by Tukey’s multiple comparisons test. *P* < 0.05 was used to determine statistical significance.

## Data Availability

Data are contained within the article.
